# Respiratory distress in the neonate: Case definition & guidelines for data collection, analysis, and presentation of maternal immunization safety data

**DOI:** 10.1016/j.vaccine.2017.01.046

**Published:** 2017-12-04

**Authors:** Leigh R. Sweet, Cheryl Keech, Nicola P. Klein, Helen S. Marshall, Beckie N. Tagbo, David Quine, Pawandeep Kaur, Ilia Tikhonov, Muhammad Imran Nisar, Sonali Kochhar, Flor M. Muñoz, For the Brighton Collaboration Respiratory Distress in the Neonate Working Group

**Affiliations:** a St. Mary’s Regional Medical Center, United States; b Pharmaceutical Product Development, United States; c Kaiser Permanente Vaccine Study Center, United States; d Women’s and Children’s Health Network and Robinson Research Institute and School of Medicine, University of Adelaide, South Australia, Australia; e Institute of Child Health & Department of Paediatrics, University of Nigeria Teaching Hospital, Nigeria; f Simpson’s Centre for Reproductive Health, Royal Infirmary Edinburgh, Scotland, United Kingdom; g Clinical Development Services Agency, India; h Sanofi Pasteur, United States; i Aga Khan University, Pakistan; j Global Healthcare Consulting, India; k Baylor College of Medicine, United States; l Erasmus University Medical Center, Rotterdam, The Netherlands

**Keywords:** Respiratory distress, Difficulty breathing, Neonate, Newborn, Adverse event, Maternal immunization, Guidelines, Case definition, Brighton Collaboration, GAIA

## 1 Preamble

### 1.1 Need for developing case definitions and guidelines for data Collection, Analysis, and presentation for respiratory distress in the neonate as an adverse event following maternal immunization

**Definition of respiratory distress in the neonate**

Every year, an estimated 2.9 million babies die in the neonatal period (the first 28 days of life), accounting for more than half of the under-five child deaths in most regions of the world, and 44% globally [1]. The majority (∼75%) of these deaths occur in the first week of life, with the highest risk of mortality concentrated in the first day of life [2]. Ninety-nine percent of neonatal deaths occur in low- and middle-income countries; south-central Asian countries experience the highest absolute numbers of neonatal deaths, while countries in sub-Saharan Africa generally have the highest rates of neonatal mortality [2].

Respiratory distress is one of the most common problems neonates encounter within the first few days of life [3]. According to the American Academy of Pediatrics, approximately 10% of neonates need some assistance to begin breathing at birth, with up to 1% requiring extensive resuscitation [4]. Other reports confirm that respiratory distress is common in neonates and occurs in approximately 7% of babies during the neonatal period [3], [5]. Respiratory disorders are the leading cause of early neonatal mortality (0–7 days of age) [6], as well as the leading cause of morbidity in newborns [7], and are the most frequent cause of admission to the special care nursery for both term and preterm infants [8]. In fact, neonates with respiratory distress are 2–4 times more likely to die than neonates without respiratory distress [9].

Respiratory distress describes a symptom complex representing a heterogeneous group of illnesses [3]. As such, respiratory distress is often defined as a clinical picture based on observed signs and symptoms irrespective of etiology [7], [10]. Clinical symptoms most commonly cited as indicators of respiratory distress include tachypnea [3], [7], [8], [10], [11], [12], [13], [14], [15], [16], [17], nasal flaring [3], [7], [8], [10], [11], [12], [13], [14], [15], [17], grunting [3], [7], [8], [10], [11], [12], [13], [14], [15], [16], [17], retractions [3], [7], [8], [10], [11], [12], [13], [14], [15], [16], [17] (subcostal, intercostal, supracostal, jugular), and cyanosis [3], [7], [8], [10], [11], [13], [17]. Other symptoms include apnea [3], [8], bradypnea [8], irregular (seesaw) breathing [8], inspiratory stridor [3], [16], wheeze [16] and hypoxia [8], [14].

*Tachypnea* in the newborn is defined as a respiratory rate of more than 60 breaths per minute [12], [15], *bradypnea* is a respiratory rate of less than 30 breaths per minute, while *apnea* is a cessation of breath for at least 20 s [18]. Apnea may also be defined as cessation of breath for less than 20 s in the presence of bradycardia or cyanosis [18]. *Nasal flaring* is a compensatory symptom that is caused by contraction of alae nasi muscles, increases upper airway diameter and reduces resistance and work of breathing [8], [12], [15]. *Stridor* is a high-pitched, musical, monophonic inspiratory breath sound that indicates obstruction at the larynx, glottis, or subglottic area [15]. *Wheezing* is a high-pitched, whistling, expiratory, polyphonic sound that indicates tracheobronchial obstruction [15]. *Grunting* is an expiratory sound caused by sudden closure of the glottis during expiration in an attempt to increase airway pressure and lung volume, and to prevent alveolar atelectasis [8], [12], [15]. *Retractions* occur when lung compliance is poor or airway resistance is high, result from negative intrapleural pressure generated by contraction of the diaphragm and accessory chest wall muscles, and are clinically evident by the use of accessory muscles in the neck, rib cage, sternum, or abdomen [8], [15]. Finally, *cyanosis* is assessed by examining the oral mucosa for blue or gray discoloration and suggests inadequate gas exchange, while *hypoxemia* is signified by an oxygen saturation of less than 90% after 15 min of life [8].

**Pathophysiology of respiratory distress in the neonate**

Most causes of respiratory distress result from an inability or delayed ability of a neonate’s lungs to adapt to their new environment [14]. In utero, the lungs are fluid filled, receive less than 10–15% of the total cardiac output, and oxygenation occurs through the placenta [8], [19], [20], [21]. For the neonate to transition, effective gas exchange must be established [8], [22], alveolar spaces must be cleared of fluid and ventilated [20], [21], and pulmonary blood flow must increase to match ventilation and perfusion [14], [23]. A small proportion of alveolar fluid is cleared by Starling forces and vaginal squeeze [14], [23], however the overall process is complex, and entails rapid removal of fluid by ion transport across the airway and pulmonary epithelium [8], [20], [23]. Peak expression of these ion channels in the alveolar epithelium is achieved at term gestation, leaving preterm infants with a reduced ability to clear lung fluid after birth [14]. If ventilation or perfusion is inadequate, the neonate develops respiratory distress [14], [23].

In utero, high pulmonary vascular resistance directs blood from the right side of the heart through the ductus arteriosus into the aorta [8]. When the umbilical vessels are clamped at birth the low-resistance placental circuit is removed, systemic blood pressure is increased, and the pulmonary vasculature relaxes [8], [20]. Expansion of the lungs and increase in PaO2 results in increased pulmonary blood flow and constriction of the ductus arteriosus [8], [21]. Cardiopulmonary transition is completed after approximately 6 h [8]. The neonate’s respiratory pattern may initially be irregular, but soon becomes rhythmic at a rate of 40–60 breaths per minute [8]. A neonate’s first breaths tend to be deeper and longer than subsequent breaths [19], they are characterized by a short deep inspiration followed by a prolonged expiratory phase [24]. This breathing pattern helps the neonate develop and maintain functional residual capacity [24].

**Causes of respiratory distress in the neonate**

Respiratory distress may be the clinical presentation of numerous conditions that affect the neonate (see Table 1). Specific causes of respiratory distress may be difficult to ascertain based on clinical presentation alone. The most common causes of respiratory distress in the newborn are pulmonary in origin and include transient tachypnea of the newborn, respiratory distress syndrome, meconium aspiration syndrome, pneumonia, sepsis, pneumothorax, persistent pulmonary hypertension of the newborn, and delayed transition [13]. Extrapulmonary etiologies, such as congenital heart defects, airway malformations, inborn errors of metabolism, neurologic, and hematologic causes are less common [13].

**Table 1:** Etiologies of respiratory distress in the neonate [8], [12], [13], [15], [17].

Pulmonary	
Congenital	Pulmonary hypoplasia, congenital diaphragmatic hernia, chylothorax, pulmonary sequestration, congenital cystic adenomatous malformation of the lung, arteriovenous malformation, congenital lobar emphysema, congenital alveolar proteinosis, alveolar capillary dysplasia, congenital pulmonary lymphangiectasis, surfactant protein deficiency
Acquired	Transient tachypnea of the newborn, respiratory distress syndrome, meconium aspiration syndrome, pneumonia, pneumothorax, pneumomediastinum, atelectasis, pulmonary hemorrhage, bronchopulmonary dysplasia, persistent pulmonary hypertension of the newborn, diaphragmatic paralysis, drug reaction, anaphylactic reaction, hypersensitivity syndrome, inhalation exposure

Extrapulmonary	

Airway	Nasal obstruction, choanal atresia, nasal stenosis, micrognathia, Pierre Robin anomaly, cleft palate, macroglossia, glossoptosis, laryngeal stenosis or atresia, tracheal atresia, laryngeal cyst or web, vocal cord paralysis, subglottic stenosis, hemangioma, papilloma, laryngomalacia, tracheobronchomalacia, tracheobronchial stenosis, tracheoesophageal fistula, vascular rings, cystic hygroma and external compression from other neck masses
Cardiovascular	Transposition of the great arteries, tetralogy of fallot, large septal defects, patent ductus arteriosus, coarctation of the aorta, congestive heart failure, cardiomyopathy, pneumopericardium
Hematologic	Polycythemia, anemia, severe hemolytic disease, hypovolemia, hereditary hemoglobinopathies, hereditary methemoglobinemia
Infectious	Sepsis, bacteremia, meningitis
Metabolic	Hypoglycemia, hypocalcemia, hypermagnesemia, hypo- or hypernatremia, inborn errors of metabolism
Neuromuscular	Hypoxic-ischemic encephalopathy, intracranial hemorrhage, hydrocephalus, seizure, narcotic withdrawal, muscle and spinal cord disorders
Thoracic	Skeletal dysplasias
Miscellaneous	Asphyxia, acidosis, hypothermia, hyperthermia, hydrops fetalis

*Transient Tachypnea of the Neonate* (*TTN*) is the most common etiology of respiratory distress in the neonatal period [8], [13]. TTN occurs in near-term, term and late preterm infants, and affects 3.6–5.7 per 1000 term infants, and up to 10 per 1000 preterm infants [8], [17]. TTN is a result of delayed resorption and clearance of alveolar fluid from the lungs [5], [13]. Following delivery, the release of prostaglandins distends lymphatic vessels which remove lung fluid as pulmonary circulation increases following the first fetal breath [13]. Cesarean section prior to the onset of labor bypasses this process, and is therefore a risk factor for TTN [8], [13], [17]. Other risk factors include surfactant deficiency [13], maternal asthma, diabetes, prolonged labor, and fetal distress requiring maternal anesthesia or analgesia [8], [17], [25]. TTN presents within the first two hours after birth and can persist for up to 72 h [13]. Clinical presentation includes rapid, shallow breathing with occasional grunting or nasal flaring [17], and rarely respiratory failure [8]. Breath sounds may either be clear, or reveal rales on auscultation [13]. TTN is generally a self-limited disorder [5], however, the higher the initial respiratory rate, the longer TTN is likely to last [13].

*Respiratory Distress Syndrome* (*RDS*) is seen soon after birth, and worsens during the first few hours of life [8], [17]. RDS occurs because of surfactant deficiency or dysfunction resulting in increased alveolar surface tension and alveolar collapse at the end of expiration [8], [17]. The disease progresses rapidly [13], with increased work of breathing, intrapulmonary shunting, ventilation perfusion mismatch, and hypoxia with eventual respiratory failure [8], [17]. The risk of RDS is inversely proportional to gestational age; RDS occurs in approximately 5% of near-term infants, 30% of infants less than 30 weeks gestational age, and 60% of premature infants less than 28 weeks gestational age [8], [17]. Additional factors associated with development of RDS are male sex in Caucasians, infants born to mothers with diabetes, perinatal asphyxia, hypothermia, multiple gestations, cesarean delivery without labor, and presence of RDS in a previous sibling [8], [17], [25]. Symptoms include tachypnea, grunting, retractions and cyanosis [8], [13].

*Meconium Aspiration Syndrome* (*MAS*) occurs in term or post-term infants born through meconium-stained amniotic fluid [17], and is seen within a few hours after birth [8]. Although meconium-stained amniotic fluid is present in 10–15% of deliveries, most infants born to mothers with meconium-stained amniotic fluid are asymptomatic, and the incidence of MAS is only 1% [8], [13]. Meconium excretion is representative of fetal maturity, therefore MAS is most commonly seen in term and post-term neonates [13]. Meconium is passed in utero when the fetus is distressed and relaxes the anal sphincter [17]. The resultant hypoxia and subsequent gasping lead to aspiration of meconium before birth [5], [8]. Meconium consists of desquamated cells, skin, lanugo hair, vernix, bile salts, pancreatic enzymes, lipids, mucopolysaccharides, and water [8], [17]. Chemical pneumonitis occurs when bile salts and other components of meconium deactivate pulmonary surfactant resulting in atelectasis [8]. Meconium also activates the complement cascade, causing inflammation and constriction pulmonary veins [8], [17]. Risk factors include preeclampsia, maternal diabetes, chorioamnionitis, and illicit substance abuse [8]. MAS presents with tachypnea, grunting, retractions and cyanosis [13]. Affected neonates may have a barrel-shaped chest, rales and rhonchi heard on auscultation, and meconium staining of the nails and umbilical cord [8], [13], [17].

*Pneumonia* is a significant cause of respiratory distress in the neonate and may be classified as early-onset (less than or equal to 7 days of age) or late-onset (greater than 7 days of age) [8]. Early-onset pneumonia most commonly occurs within the first three days of life, and is the result of placental transmission of bacteria or aspiration of infected amniotic fluid, while late-onset pneumonia occurs after hospital discharge and community exposure, resulting in various potential etiologies including viral and bacterial pathogens [13]. The clinical signs in neonatal pneumonia mimic other conditions like TTN, RDS or MAS, making it difficult to distinguish them [5], [8], [17].

**Assessment of respiratory distress in the neonate**

Initial assessment of an infant with respiratory distress should focus on the physical examination and rapid identification of life-threatening conditions [8], [17]. Assessment for respiratory distress may differ depending on clinical setting but should include at least some of the following parameters: (1) measurement of respiratory rate (normal 40–60); (2) observation for increased work of breathing: inspiratory sternal, intercostal and subcostal recession/in-drawing, tracheal tug; (3) assessment for airway noises such as expiratory grunting or inspiratory stridor; (4) assessment for nasal flaring or head bobbing; (5) assessment of color for cyanosis, ideally pulse oximetry measurement should be obtained if any concern about color/cyanosis. Apnea should prompt urgent medical assessment. Respiratory distress may be accompanied by increased, decreased, or normal respirations depending on the level of respiratory fatigue the infant is experiencing. Therefore, respiratory rate alone may not be indicative of the degree of distress. Utilizing a validated scoring system can improve the predictive value of the degree of respiratory distress and aid the practitioner in accessing additional support services in a timely fashion.

If providers are able to identify signs of respiratory distress prior to the onset of refractory disease, this may facilitate early intervention, and reduced morbidity and mortality [11]. Early warning tools may aid in the early identification of neonates at risk for clinical deterioration. These tools may also provide a standardized observation chart for monitoring clinical progress, and provide visual prompts to aid identification of abnormal parameters. Early identification of ill neonates and early intervention may facilitate early transfer to higher level care if necessary and available [26].

Several scoring systems focused specifically on assessment of respiratory distress in the neonate are available. The World Health Organization provides the most simplified scoring system, which classifies breathing difficulty based on respiratory rate, grunting and chest in-drawing [27] (see Appendix A). Other respiratory specific scoring systems include the ACoRN (Acute Care of at-Risk Newborns) Respiratory Score [11], the Silverman Scoring System [15], [28], [29], and the Downes Respiratory Distress Score (Downes RDS) [15], [30] (see Appendix A and Table 2). These respiratory specific scoring systems are based on clinical criteria, and therefore can be implemented in most settings.

**Table 2:** Comparison of validated neonatal scoring system measurements.

Neonatal scoring systems						
Variable	Respiratory specific			General neonatal illness		
	ACorN	Silverman	Downes	SNS	SNAP-II	NTS
Time dependent assessment	NA	NA	NA	NA	Yes, over 12 h	Yes, over 12 h
Respiratory rate (breaths/min, apnea)	Yes	NA	Yes	Yes	NA	Yes
Nasal flaring	NA	NA	NA	NA	NA	Yes, as components of ‘respiratory distress’
Grunting	Yes	Yes	Yes	Yes	NA	
Intercostal retractions	Yes	Yes	Yes	NA	NA	
Cyanosis	NA	NA	Yes	NA	NA	
Mean blood pressure	NA	NA	NA	Yes	Yes	NA
Oxygen measurement or requirement	Yes	NA	NA	Yes, SpO_2_ (room air)	Yes, PO_2_/FiO_2_ and pH (blood gas)	NA
Temperature	NA	NA	NA	Yes	Yes	Yes
Heart rate	NA	NA	NA	Yes	NA	Yes
Blood sugar	NA	NA	NA	Yes	NA	Yes^a^
Urine output	NA	NA	NA	NA	Yes	NA
Neurologic	NA	NA	NA	NA	Yes, seizure	Yes, level of conscious
Breath sounds on auscultation	Yes	NA	Yes	NA	NA	NA
Other	Prematurity	Paradoxic chest and abdominal movements (see-saw respirations)	NA	Capillary filling time (sec)	NA	NA
		Xiphoid retraction				
		Chin descending with respirations				

a If indicated by past history.

In addition to respiratory specific scoring systems, there are also general neonatal illness scoring systems. These include the Sick Neonate Score (SNS) [31], the Score for Neonatal Acute Physiology II (SNAP-II) [32], and the Neonatal Trigger Score (NTS) [33] (see Appendix B and Table 2). Although by definition these scores are more representative of overall neonatal illness, each does take respiratory symptoms into account, and therefore may also help determine the presence of respiratory distress in the neonate. SNS is a clinical score that was developed to assess neonatal illness in resource limited settings [31]. SNAP-II and NTS require 12 h of data collection, and SNAP-II requires assessment of urine output and a blood gas, which may make it more difficult to implement these scoring systems in some settings [11], [32], [33].

**Respiratory Distress in the Neonate following maternal immunization**

Influenza vaccine is recommended for pregnant women in many countries at any time during pregnancy to prevent infection in both the pregnant woman and her neonate [34]. The safety of influenza vaccine during pregnancy has been studied with no evidence of safety concerns when administered in any trimester [34], [35], [36]. Although three systematic reviews have supported the evidence for no safety signal, there are limitations on the amount of evidence available, especially for more specific pregnancy outcomes such as congenital malformations, in women receiving influenza vaccine in the first trimester [35]. Respiratory symptoms in the neonate following maternal immunization are rarely reported [37]. In a large retrospective database review over 5 influenza seasons, Muñoz et al. reported on “respiratory problems” in the neonate within 2 days of birth. No infants had respiratory problems if their mother had received influenza vaccine during pregnancy, compared to 8 infants with respiratory problems whose mother had not received influenza vaccine, however this difference was not statistically significant (p = 0.2) [38].

The evaluation of low APGAR scores (<7) as an adverse event following maternal influenza immunization, and which includes an assessment of respiratory effort, has been reported in six studies [39], [40], [41], [42], [43], [44]. These studies mostly relate to pandemic influenza vaccine (influenza A H1N1 09 vaccine) with one reporting on influenza A Hsw1N1 vaccine [39]. Only the study by Håberg et al. had a point estimate that favored the unvaccinated cohort, although this was close to the null value and did not reach statistical significance (HR = 1.08 (95% CI, 0.91–1.28) [41]. The remainder of the cohort studies had a point estimate that favored the vaccinated cohort. A prospective cohort study reported an unadjusted OR = 0.88 (CI 95% 0.35–2.20) and a retrospective cohort study reported a RR = 0.97 (95% CI 0.82, 1.14) for APGAR < 7 [39], [40]. A cross-sectional study indicated a protective effect against 5 min APGAR score <7, unadjusted OR = 0.7 (95% CI 0.47–1.05) [44]. None of the studies demonstrated any statistical or clinical association with decreased APGAR scores.

Pertussis-containing vaccines used in pregnant women often contain tetanus toxoid, diphtheria toxoid, acellular pertussis, and inactivated poliomyelitis antigens (Tdap or Tdap-IPV). In pregnant women, administration of a lower antigen pertussis-containing vaccine is recommended during the third trimester of pregnancy (or earlier in some countries), to ensure maximal and timely protection for neonates [45], [46]. Large cohort studies examining the safety of Tdap/Tdap-IPV vaccine administered in pregnancy have not identified any safety concerns [47], [48], [49], [50], [51], [52]. Morgan et al. provide the only published data on respiratory outcomes in neonates in pregnant women who have received Tdap vaccine. In this retrospective cohort study comparing women who did and did not receive Tdap vaccine in pregnancy, no difference was observed in infants with a 5-min APGAR score <4 [48]. No difference was observed between these groups in neonatal complications, including requirement for ventilation in the first 24 h. A subgroup analysis of multiparous women who received at least 2 doses of Tdap vaccine in the past 5 years compared to one dose of Tdap demonstrated comparable neonatal outcomes, including ventilation requirements [48].

**Existing case definitions for respiratory distress in the neonate**

Respiratory distress in the newborn is a common clinical syndrome with many possible etiologies. Several definitions of respiratory distress are currently available from a variety of organizations and in the literature. These are summarized in Table 3. If not cited, no specific definition was identified from certain organizations (e.g. American Academy of Pediatrics, CIOMS, MedDRA).

**Table 3:** Existing case definitions of respiratory distress in the neonate.

Source	Definition
World Health Organization	Respiratory rate more than 60 or less than 30 breaths per minute, grunting on expiration, chest indrawing, or central cyanosis [blue tongue and lips], apnoea (spontaneous cessation of breathing for more than 20 s)
NCI/NICHD	Increased work of breathing with tachypnea and retractions
2016 ICD-10 CM diagnosis code	A condition of the newborn marked by dyspnea with cyanosis, heralded by such prodromal signs as dilatation of the alae nasi, expiratory grunt, and retraction of the suprasternal notch or costal margins, most frequently occurring in premature infants, children of diabetic mothers, and infants delivered by cesarean section, and sometimes with no apparent cause
Kumar M, et al. Arch Dis Child Fetal Neonatal Ed 2014;99:F116.	One or more of the following: need for supplemental oxygen > or = 2 h and/or positive pressure ventilation (CPAP or endotracheal intubation) following admission to neonatal intensive care unit
Swiss Society of Neonatology Definition in Ersch et al. Acta Paediatrica 2007;96:1577	Presence of at least two of the following criteria: tachypnea (>60 breaths per minute), central cyanosis in room air, expiratory grunting, subcostal, intercostal or jugular retractions and nasal flaring. Entirely based on clinical observation irrespective of etiology
Ma X, et al. Chin Med J 2010;123(20):2777	Clinical signs of effort breathing, such as tachypnea, grunting, intercostal retraction, nasal flaring and cyanosis
Qian L, et al. Chin Med J 2010;123(20):2770	At least two of the following criteria: tachypnea, central cyanosis in room air, expiratory grunting, sub-costal, intercostals or jugular retractions and nasal flaring. Entirely based on clinical observation irrespective of etiology
Pramanik AK, et al. Pediatr Clin N Am 2015;62:454–55	Tachypnea (rate >60 breaths per minute), cyanosis, expiratory grunting with chest retractions, and nasal flaring. Decrease in oxygen saturation, apnea or both may be present. Irregular (seesaw) or slow respiratory rates of less than 30 breaths per minute if associated with gasping may be an ominous sign
Hermansen CL, et al. Am Fam Physician 2015;92(11):994	Tachypnea is most common presentation. Other signs may include nasal flaring, grunting, intercostal or subcostal retractions, and cyanosis
Parkash A, et al. JPMA 2015;65:771	Presence of one or more of the following clinical features: respiratory rate >60 breaths/minute, chest wall retraction, grunting, nasal flaring and cyanosis
Mahoney AD, et al. Clin Perinatol 2013;40:666	Sustained distress for more than 2 h after birth accompanied by grunting, flaring, tachypnea, retractions, or supplemental oxygen requirement
Reuter S, et al. Ped Rev 2014;35(10):418	Recognized as one or more signs of increased work of breathing, such as tachypnea, nasal flaring, chest retractions, or grunting
Hermansen CL, et al. Am Fam Physician 2007;76:987	The clinical presentation includes apnea, cyanosis, grunting, inspiratory stridor, nasal flaring, poor feeding, and tachypnea (more than 60 breaths per minute). There may also be retractions in the intercostal, subcostal, or supracostal spaces
Warren JB, et al. Pediatr Rev 2010;31(12):487–95	Most commonly presents as one or all of the following physical signs: tachypnea, grunting, nasal flaring, retractions and cyanosis
Edwards MO, et al. Pediatric Respiratory Reviews 2013;14:30	Recognized as any signs of breathing difficulties in the neonate. Tachypnea (RR > 60/min) & tachycardia (HR > 160/min, cyanosis, nasal flaring, grunting, apnoea/dyspnoea, chest wall recessions (suprasternal, intercostal & subcostal)
Mathai SS, et al. MJAFI 2007;63:269	Diagnosed when one or more of the following is present: tachypnoea or respiratory rate of more than 60/min, retractions or increased chest in drawings on respirations (subcostal, intercostal, sternal, suprasternal) and noisy respiration in the form of a grunt, stridor, or wheeze. The distress may or may not be associated with cyanosis and desaturation on pulse oximetry

**Need for a harmonized definition of respiratory distress in the neonate**

There is no uniformly accepted case definition of Respiratory Distress in the Neonate in the context of assessing adverse events following maternal immunization. There is variability in existing definitions, which decreases their specificity. Data comparability across trials or surveillance systems would facilitate data interpretation, improve harmonization across clinical and population studies, and promote the scientific understanding of Respiratory Distress in the Neonate.

### 1.2 Methods for the development of the case definition and guidelines for data collection, analysis, and presentation for respiratory distress in the neonate as an adverse event following maternal immunization

Following the process described in the overview papers [53], [54] as well as on the Brighton Collaboration Website http://www.brightoncollaboration.org/internet/en/index/process.html, the Brighton Collaboration *Respiratory Distress in the Neonate Working Group* was formed in 2016 and included members of various clinical, academic, public health, and industry backgrounds. The composition of the working and reference group as well as results of the web-based survey completed by the reference group with subsequent discussions in the working group can be viewed at: http://www.brightoncollaboration.org/internet/en/index/working_groups.html.

To guide the decision-making for the case definition and guidelines, literature searches were performed using PubMed, Medline, Embase, Clinical Key and the Cochrane Libraries. One literature search focused on general descriptions of respiratory distress in the neonate, was conducted using PubMed, searched English language articles only, and used the search terms “respiratory distress” and “neonate”. The search resulted in 4000 articles from 2006 to present, all titles and abstracts were reviewed. Fifty-four articles with potentially relevant material were reviewed in full to identify case definitions, background rates, etiologies and pathophysiology of respiratory distress in the neonate.

Of the 54 articles reviewed on respiratory distress and the neonate, 33 were relevant, and a total of 16 definitions of respiratory distress in the neonate were identified (Table 3). These case definitions were noted to contain similar elements, but there was variation in terminology used, number and type of symptoms considered, and application of the definition. An inventory of the 16 relevant case definitions of Respiratory Distress in the Neonate was made available to working group members.

An additional search was conducted to identify literature about maternal immunization in relation to respiratory distress in the neonate. This search utilized the terms “maternal immunization, vaccine, vaccines, vaccination, immunization, pregnancy, neonatal, neonate, newborn, infant, respiratory distress, respiratory insufficiency, apnoea, apnea, apneic attack, apnoeic attack, respiratory arrest, respiratory failure, respiratory acidosis, respiratory complications, difficulty breathing, increased work of breathing, labored respiration, pneumonia, pulmonary, respiratory tract, pulmonary edema, pulmonary oedema, alveolitis, lung infiltration, interstitial lung disease”. The search was limited to publications from 2005 to present, and concentrated on reviews or large clinical studies*.* The search resulted in the identification of 56 references, 9 of which were book chapters. All English language article abstracts were screened for possible reports of Respiratory Distress in the Neonate following maternal immunization. Forty-seven articles with potentially relevant material were reviewed in more detail, in order to identify studies using case definitions or, in their absence, providing clinical descriptions of the case material. This review resulted in a detailed summary of 47 articles, including information on the study type, the vaccine, the diagnostic criteria or case definition put forth, the time interval since time of immunization, and any other symptoms.

Of the 47 articles reviewed on immunization and respiratory distress, 17 focused on maternal immunization, while 30 focused on infant immunization. Most of the papers on maternal immunization did not mention Respiratory Distress in the Neonate as an adverse event that was considered in relation to maternal immunization. When respiratory distress was mentioned in a few of these articles it was not clearly defined. The 30 articles related to infant immunization were not relevant, as they focused on infants immunized outside of the neonatal age range, and were not related to maternal immunization.

### 1.3 Rationale for selected decisions about the case definition of respiratory distress in the neonate as an adverse event following maternal immunization

The term Respiratory Distress in the Neonate refers to a constellation of clinical findings that support the presence of breathing difficulty in the neonate (0 to 28 days of life), independent from etiology or severity, and independent from the infant’s gestational age or circumstances at the time of delivery. Respiratory distress is distinct from the clinical findings observed during normal transition from intra- to extra- uterine life in all newborns. Different terminology exists in the literature in relation to respiratory distress in the neonate, from a very broad characterization as “increased work of breathing” or “dyspnea”, to various measurable findings (e.g. respiratory rate), to observing for the presence of clinical findings consistent with difficulty breathing (e.g. expiratory grunting, chest retractions) or with the consequences of poor oxygenation (e.g. central cyanosis), to, in some cases, laboratory findings (e.g. arterial blood gas analysis).

Different terminologies in the literature that refer to the clinical syndrome of Respiratory Distress in the Neonate were identified, including: respiratory distress, difficulty breathing, labored breathing, shortness of breath, increased work of breathing, labored respirations, respiratory insufficiency, respiratory failure, respiratory arrest, respiratory acidosis, respiratory complications, respiratory disease, respiratory illness, and respiratory disorder. The term Respiratory Distress Syndrome is utilized specifically to designate hyaline membrane disease, and it is distinct from the term Respiratory Distress in the Neonate selected for this case definition.

Numerous related term(s) of Respiratory Distress in the Neonate exist in the literature. Some have the observed clinical findings associated with respiratory distress in neonates (e.g. apnea, apneic attack, bradypnea, tachypnea, dyspnea, retractions, recessions, use of accessory muscles, cyanosis, grunting, stridor, nasal flaring, wheezing), while others reflect the possible etiologies of respiratory distress (e.g. Respiratory Distress Syndrome, hyaline membrane disease, surfactant deficiency lung disease, meconium aspiration syndrome, transient tachypnea of the newborn, persistent pulmonary hypertension of newborn, hypoxia, pneumonia, pulmonary edema, alveolitis, lung infiltration, interstitial lung disease).

Disparity in the use of respiratory distress during the neonatal period may result in inconsistent classifications for adverse event reporting. It is important to highlight that when choosing to report on an adverse event associated with vaccination, the most precise definition or description of the event should be cataloged. Therefore, although respiratory distress may often present as a symptom of a disease, the more precise disease etiology should be the term chosen for the adverse event (e.g. meconium aspiration would be more precise than respiratory distress, although both would be present for the single situation).

**Focus of brighton collaboration case definition**

The focus of the Working Group was to identify all the necessary components to define Respiratory Distress in the Neonate, and to produce a harmonized definition to properly identify cases of respiratory distress in the neonate in the context of vaccination of mothers during pregnancy. Within the definition context, however, the three diagnostic levels must not be misunderstood as reflecting different grades of clinical severity. They instead reflect diagnostic certainty (see below). Furthermore, the definition may be applied to settings other than studies of vaccines in pregnancy.

The Brighton Collaboration case definition of respiratory distress in the neonate is based on clinical observation only, utilizing auscultation with stethoscope when available. However, supporting evidence from certain devices may be utilized in certain clinical settings, such as pulse oximetry or a cardiac and respiratory monitor. The definition based on clinical criteria is applicable in different settings, independent from resources. However, collection of additional information based on laboratory, imaging, or pathology results is encouraged to ascertain the cause of the syndrome manifesting as respiratory distress in the newborn. A summary of potential etiologies is described in Table 1.

**Formulating a case definition that reflects diagnostic certainty: weighing specificity versus sensitivity**

It needs to be re-emphasized that the grading of definition levels is entirely about diagnostic certainty, not clinical severity of an event. Thus, a clinically very severe event may appropriately be classified as Level Two or Three rather than Level One if it could not reasonably be confirmed to fit within the case definition of Respiratory Distress in the Neonate. Detailed information about the severity of the event should additionally always be recorded, as specified by the data collection guidelines.

The number of symptoms and/or signs that will be documented for each case may vary considerably. The case definition has been formulated such that the Level 1 definition is highly specific for the condition. As maximum specificity normally implies a loss of sensitivity, two additional diagnostic levels have been included in the definition, offering a stepwise increase of sensitivity from Level One down to Level Three, while retaining an acceptable level of specificity at all levels. In this way it is hoped that all possible cases of Respiratory Distress in the Neonate can be captured.

**The meaning of “Sudden Onset” and “Rapid progression” in the context of respiratory distress in the neonate**

The term “sudden onset” refers to an event that occurred unexpectedly and without warning leading to a marked change in a subject’s previously stable condition.

The term “rapid progression” is a conventional clinical term. Respiratory distress may be classified as occurring unexpectedly and of being of “sudden onset”, and clinical progression can be assesses as “rapid” by the provider. An exact time-frame of what rapid progression is should not be offered since progression may be associated with wide range of potential etiologies. Documentation of these characteristics however, should be helpful during the evaluation of respiratory distress in the neonate, in order to correlate with potential etiologies and interventions.

**Rationale for individual criteria or decision made related to the case definition**

**Pathology findings**

Pathology is not necessary for the ascertainment of respiratory distress in the neonate, given that the diagnosis of respiratory distress is based on clinical observation. However, pathology findings are helpful for the identification of etiologic causes of respiratory distress in the newborn.

**Radiology findings**

Radiology findings are not necessary for the ascertainment of respiratory distress in the neonate, given that the diagnosis of respiratory distress is based on clinical observation. However, radiology findings are helpful for the identification of etiologic causes of respiratory distress in the newborn, specifically for the identification of pulmonary vs. extrapulmonary causes of respiratory distress.

**Laboratory findings**

Laboratory findings are not necessary for the ascertainment of respiratory distress in the neonate, given that the diagnosis of respiratory distress is based on clinical observation. However, laboratory findings are helpful for the identification of etiologic causes of respiratory distress in the newborn. For example, the result of arterial or venous blood gas analysis can confirm the presence of hypoxemia, and the presence of leukocytosis or a positive blood culture can identify an infectious etiology for respiratory distress.

**Influence of treatment on fulfillment of case definition**

The Working Group decided against using “treatment” or “treatment response” towards fulfillment of the Respiratory Distress in the Neonate case definition.

A treatment response or its failure is not in itself diagnostic, and may depend on variables like clinical status, time to treatment, and other clinical parameters.

An important consideration is that practically all newborns will require some form of reanimation after delivery (e.g. stimulation, suctioning of secretions, blow by oxygen, etc.), and that infants may present with clinical findings at birth that could be considered part of the clinical manifestations of Respiratory Distress (e.g. tachypnea, bradypnea, apnea, nasal flaring, retractions and cyanosis). However, these routine clinical findings and interventions should NOT be considered for the fulfillment of the case definition of respiratory distress in the neonate if they occur and then dissipate with standard delivery/post-delivery care in the first 10 min of life. Interventions that are beyond routine neonatal reanimation at birth needed to support an infant who meets the case definition of respiratory distress, should be documented.

**Timing post maternal immunization**

Specific time frames for onset of Respiratory Distress in the Neonate following maternal immunization are not included as a consideration when ascertaining the case definition. By our definition Respiratory Distress in the Neonate occurs after delivery to any time in the first 28 days of the infant’s life. The time interval between maternal immunization and delivery is variable depending on the study design and other events of pregnancy.

We postulate that a definition designed to be a suitable tool for testing causal relationships requires ascertainment of the outcome (e.g. Respiratory Distress in the Neonate) independent from the exposure (e.g. maternal immunizations). Therefore, to avoid selection bias, a restrictive time interval from maternal immunization to onset of Respiratory Distress in the Neonate should not be an integral part of such a definition. Instead, where feasible, details of this interval should be assessed and reported as described in the data collection guidelines.

Further, Respiratory Distress in the Neonate may occur outside the controlled setting of a clinical trial or hospital. In some settings it may be impossible to obtain a clear timeline of the event, particularly in less developed or rural settings. In order to avoid selecting against such cases, the Brighton Collaboration case definition avoids setting arbitrary time frames.

**Differentiation from other (similar/associated) disorders**

Respiratory Distress in the Neonate is distinct from normal signs and symptoms of transition to extrauterine life occurring immediately after delivery. These are transient and not associated with any pathology, typically resolving after stimulation and not requiring specific treatment. It is also distinct from Respiratory Distress Syndrome (RDS), a term used to describe a very specific condition, also known as surfactant deficiency or hyaline membrane disease of the newborn. See more detailed description in Section 1.1.

### 1.4 Guidelines for data collection, analysis and presentation

As mentioned in the overview paper, the case definition is accompanied by guidelines which are structured according to the steps of conducting a clinical trial or conducting vaccine safety monitoring, i.e. data collection, analysis and presentation. Neither case definition nor guidelines are intended to guide or establish criteria for management of ill infants, children, or adults. Both were developed to improve data comparability.

### 1.5 Periodic review

Similar to all Brighton Collaboration case definitions and guidelines, review of the definition with its guidelines is planned on a regular basis (i.e. every three to five years) or more often if needed.

## 2 Case definition of respiratory distress in the neonate

**For All Levels of Diagnostic Certainty**

**Respiratory Distress in the Neonate** is a **clinical syndrome** occurring in **Newborns 0 to 28 days of life**, characterized by the presence of:

**Abnormal respiratory rate**

Measurement of number of breaths per minute consistent with:

Tachypnea = respiratory rate of 60 or more breaths per minute
OR
Bradypnea = respiratory rate of less than 30 breaths per minute
OR
Apnea = cessation of respiratory effort (no breaths) for at least 20 s
**AND**

**Clinical symptoms consistent with labored breathing**

Clinical observation of:

Nasal flaring (dilatation of alae nasi)
OR
Noisy respirations in the form of expiratory grunting, stridor, or wheeze
OR
Retractions or increased chest indrawings on respiration (subcostal, intercostal, sternal, suprasternal notch)
OR
Central cyanosis (whole body, including lips and tongue) on room air
OR
Low Apgar Score (<7 points) at 10 min, with respiration score <2

The ascertainment of respiratory distress in the neonate is independent from the newborn’s gestational age at the time of delivery and the circumstances of delivery, and distinct from the clinical manifestations of the immediate normal transition from intrauterine to extrauterine life. Clinical findings should therefore be persistent beyond the first 10 min of life (when Apgar scores are collected), or occur at any time after this transition period and before day of life 28. Clinical findings consistent with respiratory distress should be assessed prior to any intervention or assistance needed in response to the findings. Ascertainment of the diagnosis is not dependent on the need for, or results of, medical interventions or the type of intervention initiated (e.g. need for supplemental oxygen, positive pressure support, or mechanical ventilation). Provision of respiratory support (e.g. airway placement, oxygen supplementation) in itself is not always indicative of Respiratory Distress in the Neonate. Furthermore, the absence of an abnormal respiratory rate does not rule out the diagnosis of respiratory distress in infants who have had an abnormal respiratory rate, transiently appear normal, and continue to deteriorate. The case definition identifies cases of respiratory distress in the neonate, independently from the cause or the severity of the clinical findings of respiratory distress.

Additional supporting evidence of respiratory distress (but not required for case ascertainment) may include: Hypoxemia documented by pulse oximetry or arterial or venous blood gas analysis, presence of tachycardia or bradycardia, decreased muscular tone, flaccid/limp muscles, body or extremities, hypo-responsiveness, and obtundation.

**Diagnostic levels of certainty**

**Level 1**

Newborn 0 to 28 days of life
**AND**
**Abnormal respiratory rate**
Measurement of number of breaths per minute consistent with:
Tachypnea = respiratory rate of 60 or more breaths per minute
OR
Bradypnea = respiratory rate of less than 30 breaths per minute
OR
Apnea = cessation of respiratory effort (no breaths) for at least 20 s
**AND**
**Clinical symptoms consistent with labored breathing**
Nasal flaring (dilatation of alae nasi)
OR
Noisy respirations in the form of expiratory grunting, stridor, or wheeze
OR
Retractions or increased chest indrawings on respiration (subcostal, intercostal, sternal, suprasternal notch)
OR
Central cyanosis (whole body, including lips and tongue) on room air
OR
Low Apgar Score (< 7 points) at 10 min, with respiration score < 2
**AND**
Examination and documentation by qualified, trained, health care provider appropriate for the clinical setting.

**Level 2**

Newborn 0 to 28 days of life
**AND**
**Abnormal respiratory rate**
Not measured, but reported as “rapid breathing”, “slow breathing”, having periods of “no breathing”, or “abnormal breathing”
**AND**
**Clinical symptoms consistent with labored breathing**
Nasal flaring (dilatation of alae nasi)
OR
Noisy respirations in the form of expiratory grunting, stridor, or wheeze
OR
Retractions or increased chest indrawings on respiration (subcostal, intercostal, sternal, suprasternal notch) or seesaw respirations
OR
Central cyanosis (whole body, including lips and tongue) on room air
OR
Low Apgar Score (<7 points) at 10 min, with respiration score <2
**AND**
No medical record documentation, but reporting through either a non-medical observer (e.g. mother, father, community worker) or via standard census mechanisms (e.g. Demographic and Health Surveillance System)
OR
Collection of information from medical record review or billing codes.

**Level 3**

**No need for a level 3 per working group**.

**Level 4**

**Not enough information to ascertain case of respiratory distress**.

**Level 5**

**Not a case of respiratory distress in the neonate**.

## 3 Guidelines for data collection, analysis and presentation of respiratory distress in the neonate

It was the consensus of the GAIA-Brighton Collaboration *Respiratory Distress in the Neonate Working Group* to recommend the following guidelines to enable meaningful and standardized collection, analysis, and presentation of information about Respiratory Distress in the Neonate in studies of vaccines given during pregnancy. However, implementation of all guidelines might not be possible in all settings. The availability of information may vary depending upon resources, geographical region, and whether the source of information is a prospective clinical trial, a post-marketing surveillance or epidemiological study, or an individual report of Respiratory Distress in the Neonate. Also, these guidelines have been developed by this working group for guidance only, and are not to be considered a mandatory requirement for data collection, analysis, or presentation.

### 3.1 Data collection

These guidelines represent a desirable standard for the collection of data on availability following maternal immunization to allow for comparability of data, and are recommended as an addition to data collected for the specific study question and setting. The guidelines are not intended to guide the primary reporting of Respiratory Distress in the Neonate to a surveillance system or study monitor. Investigators developing a data collection tool based on these data collection guidelines also need to refer to the criteria in the case definition, which are not repeated in these guidelines.

Guidelines number 1–43 below have been developed to address data elements for the collection of adverse event information as specified in general drug safety guidelines by the International Conference on Harmonization of Technical Requirements for Registration of Pharmaceuticals for Human Use [55], and the form for reporting of drug adverse events by the Council for International Organizations of Medical Sciences [56]. These data elements include an identifiable reporter and patient, one or more prior immunizations, and a detailed description of the adverse event, in this case, of Respiratory Distress in the Neonate following maternal immunization. The additional guidelines have been developed as guidance for the collection of additional information to allow for a more comprehensive understanding of Respiratory Distress in the Neonate following maternal immunization.

#### 3.1.1 Source of information/reporter

For all cases and/or all study participants, as appropriate, the following information should be recorded:

(1) Date of report.
(2) Name and contact information of person reporting^3^ and/or diagnosing Respiratory Distress in the Neonate as specified by country-specific data protection law.
(3) Name and contact information of the investigator responsible for the subject, as applicable.
(4) Relation to the patient (e.g. immunizer [clinician, nurse], family member [indicate relationship], other).

#### 3.1.2 Vaccinee/control

##### 3.1.2.1 Demographics

For all cases and/or all study participants (mothers and infants), as appropriate, the following information should be recorded:

(5) Case/study participant identifiers (e.g. first name initial followed by last name initial) or code (or in accordance with country-specific data protection laws).
(6) Date of birth, age, and sex.
(7) For infants: Gestational age and birth weight.

##### 3.1.2.2 Clinical and immunization history

For all cases and/or all study participants (mothers and infants), as appropriate, the following information should be recorded:

(8) Past medical history, including hospitalizations, underlying diseases/disorders, pre-immunization signs and symptoms including identification of indicators for, or the absence of, a history of allergy to vaccines, vaccine components or medications; food allergy; allergic rhinitis; eczema; asthma.
(9) Any medication history (other than treatment for the event described) prior to, during, and after immunization including prescription and non-prescription medication as well as medication or treatment with long half-life or long term effect. (e.g. immunoglobulins, blood transfusion and immunosuppressants).
(10) Immunization history (i.e. previous immunizations and any adverse event following immunization (AEFI)), in particular occurrence of Respiratory Distress in the Neonate after a previous maternal immunization. Of note, ascertainment of maternal immunization history might be challenging in different settings, and collection of data from different sources might be necessary to optimize data gathering.

#### 3.1.3 Details of the immunization

For all cases and/or all study participants, as appropriate, the following information should be recorded:

(11) Date and time of maternal immunization(s).
(12) Description of vaccine(s) (name of vaccine, manufacturer, lot number, dose (e.g. 0.25 mL, 0.5 mL, etc.), name and lot number of any diluent used in the vaccine, and number of dose if part of a series of immunizations against the same disease).
(13) The anatomical sites (including left or right side) of all immunizations (e.g. vaccine A in proximal left lateral thigh, vaccine B in left deltoid).
(14) Route and method of administration (e.g. intramuscular, intradermal, subcutaneous, and needle-free (including type and size), other injection devices).
(15) Needle length and gauge.

#### 3.1.4 The adverse event

(16) For all cases at any level of diagnostic certainty and for reported events with insufficient evidence, the criteria fulfilled to meet the case definition should be recorded.
(17) Specifically document: Clinical description of signs and **symptoms of Respiratory Distress in the Neonate, and if there was medical confirmation** of the event (i.e. patient seen by physician).
(18) Date/time of onset^4^, first observation^5^ and diagnosis^6^, end of episode^7^ and final outcome.^8^
(19) Concurrent signs, symptoms, and diseases.
(20) Measurement/testing
  • Values and units of routinely measured parameters (e.g. respirations per minute, heart beats per minute, temperature) – in particular those indicating the severity of the event;
  • Method of measurement (e.g. respiratory monitor, pulse oximeter, duration of measurement, cardiac etc.);
  • Results of laboratory and radiographic examinations, surgical and/or pathological findings and diagnoses if present.
(21) Treatment given for Respiratory Distress in the Neonate
(22) Outcome^8^ at last observation.
(23) Objective clinical evidence supporting classification of the event as “serious”.^9^
(24) Exposures (e.g. food, environmental) considered potentially relevant to the reported event.

#### 3.1.5 Miscellaneous/general

(25) The duration of surveillance for Respiratory Distress in the Neonate is predefined based on the duration of the neonatal period of 28 days.
(26) The duration of follow-up reported during the surveillance period should be predefined likewise. It should aim to continue to resolution of the event.
(27) Methods of data collection should be consistent within and between study groups, if applicable.
(28) Follow-up of cases should attempt to verify and complete the information collected as outlined in data collection guidelines 1–24.
(29) Investigators of patients with Respiratory Distress in the Neonate should provide guidance to reporters to optimize the quality and completeness of information provided.
(30) Reports of Respiratory Distress in the Neonate should be collected throughout the study period regardless of the time elapsed between maternal immunization and the adverse event. If this is not feasible due to the study design, the study periods during which safety data are being collected should be clearly defined.

### 3.2 Data analysis

The following guidelines represent a desirable standard for analysis of data on Respiratory Distress in the Neonate to allow for comparability of data, and are recommended as an addition to data analyzed for the specific study question and setting.

(31) Reported events should be classified in one of the following five categories including the three levels of diagnostic certainty. Events that meet the case definition should be classified according to the levels of diagnostic certainty as specified in the case definition. Events that do not meet the case definition should be classified in the additional categories for analysis.

**Event classification in 5 categories**^10^

**Event meets case definition**

(1) Level 1: *Criteria as specified in the Respiratory Distress in the Neonate case definition*
(2) Level 2: *Criteria as specified in the Respiratory Distress in the Neonate case definition*
(3) Level 3: *Criteria as specified in the Respiratory Distress in the Neonate case definition* (*if applicable*)

**Event does not meet case definition**

***Additional categories for analysis***

(4) Reported Respiratory Distress in the Neonate with insufficient evidence to meet the case definition^11^
(5) Not a case of Respiratory Distress in the Neonate^12^
(32) The interval between maternal immunization and reported Respiratory Distress in the Neonate could be defined as the date/time of maternal immunization to the date/time of onset^4^ of the first symptoms and/or signs consistent with the definition. In this case, it is probably important to distinguish cases of Respiratory Distress occurring in the immediate post-delivery period (within 10 min), those occurring in the first week after delivery (early neonatal period or 0–6 days of life), and those occurring at or after the 7th day and up to 28 days of life (late neonatal period). Determining the interval from maternal vaccination to the event is probably more relevant for those cases occurring immediately at the time of delivery. However, in all cases, the interval between maternal vaccination(s) and the date of birth should be recorded. For a large number of cases, data could be analyzed in the following increments:

**Subjects with Respiratory Distress in the Neonate by Interval to Presentation**

**Table:** 

Interval∗	Number/percentage
Cases occuring after delivery and in the first 28 days of life	
Immediately (within 10 min) after delivery	
At 0–6 days of life	
At 7–28 days of life	

Total

(33) The duration of possible Respiratory Distress in the Neonate could be analyzed as the interval between the date/time of onset^3^ of the first symptoms and/or signs consistent with the definition and the end of episode^7^ and/or final outcome^8^. Whatever start and ending are used, they should be used consistently within and across study groups.
(34) If more than one measurement of a particular criterion is taken and recorded, the value corresponding to the greatest magnitude of the adverse experience could be used as the basis for analysis. Analysis may also include other characteristics like qualitative patterns of criteria defining the event.
(35) The distribution of data (as numerator and denominator data) could be analyzed in predefined increments (e.g. measured values, times), where applicable. Increments specified above should be used. When only a small number of cases is presented, the respective values or time course can be presented individually.
(36) Data on Respiratory Distress in the Neonate obtained from infants of subjects receiving a vaccine should be compared with those obtained from one or more appropriately selected and documented control groups to assess background rates in non-exposed populations, and should be analyzed by study arm and dose where possible, e.g. in prospective clinical trials.

### 3.3 Data presentation

These guidelines represent a desirable standard for the presentation and publication of data on Respiratory Distress in the Neonate following maternal immunization to allow for comparability of data, and are recommended as an addition to data presented for the specific study question and setting. Additionally, it is recommended to refer to existing general guidelines for the presentation and publication of randomized controlled trials, systematic reviews, and meta-analyses of observational studies in epidemiology (e.g. statements of Consolidated Standards of Reporting Trials (CONSORT), of Improving the quality of reports of meta-analyses of randomized controlled trials (QUORUM), and of meta-analysis Of Observational Studies in Epidemiology (MOOSE), respectively) [57], [58], [59].

(37) All reported events of Respiratory Distress in the Neonate should be presented according to the categories listed in guideline 32.
(38) Data on possible Respiratory Distress in the Neonate events should be presented in accordance with data collection guidelines 1–24 and data analysis guidelines 31–36.
(39) Terms to describe Respiratory Distress in the Neonate such as “mild”, “moderate”, “severe” or “significant” are highly subjective, prone to wide interpretation, and should be avoided, unless clearly defined.
(40) Data should be presented with numerator and denominator (n/N) (and not only in percentages), if available.

Although immunization safety surveillance systems denominator data are usually not readily available, attempts should be made to identify approximate denominators. The source of the denominator data should be reported and calculations of estimates be described (e.g. manufacturer data like total doses distributed, reporting through Ministry of Health, coverage/population based data, etc.).

(41) The incidence of cases in the study population should be presented and clearly identified as such in the text.
(42) If the distribution of data is skewed, median and range are usually the more appropriate statistical descriptors than a mean. However, the mean and standard deviation should also be provided.
(43) Any publication of data on Respiratory Distress in the Neonate should include a detailed description of the methods used for data collection and analysis as possible. It is essential to specify:
  • The study design;
  • The method, frequency and duration of monitoring for Respiratory Distress in the Neonate;
  • The trial profile, indicating participant flow during a study including drop-outs and withdrawals to indicate the size and nature of the respective groups under investigation;
  • The type of surveillance (e.g. passive or active surveillance);
  • The characteristics of the surveillance system (e.g. population served, mode of report solicitation);
  • The search strategy in surveillance databases;
  • Comparison group(s), if used for analysis;
  • The instrument of data collection (e.g. standardized questionnaire, diary card, report form);
  • Whether the day of immunization was considered “day one” or “day zero” in the analysis;
  • Whether the date of onset^4^ and/or the date of first observation^5^ and/or the date of diagnosis^6^ was used for analysis; and
  • Use of this case definition for Respiratory Distress in the Neonate, in the abstract or methods section of a publication.^13^

## Disclaimer

The findings, opinions and assertions contained in this consensus document are those of the individual scientific professional members of the working group. They do not necessarily represent the official positions of each participant’s organization (e.g., government, university, or corporation). Specifically, the findings and conclusions in this paper are those of the authors and do not necessarily represent the views of their respective institutions.

## Footnotes

3 If the reporting center is different from the vaccinating center, appropriate and timely communication of the adverse event should occur.

4 The date and/or time of onset is defined as the time post immunization, when the first sign or symptom indicative of Respiratory Distress in the Neonate occurred. This may only be possible to determine in retrospect.

5 The date and/or time of first observation of the first sign or symptom indicative of Respiratory Distress in the Neonate can be used if date/time of onset is not known.

6 The date of diagnosis of an episode is the day post immunization when the event met the case definition at any level.

7 The end of an episode is defined as the time the event no longer meets the case definition at the lowest level of the definition.

8 E.g. recovery to pre-immunization health status, spontaneous resolution, therapeutic intervention, persistence of the event, sequelae, death.

9 An AEFI is defined as serious by international standards if it meets one or more of the following criteria: 1) it results in death, 2) is life-threatening, 3) it requires inpatient hospitalization or results in prolongation of existing hospitalization, 4) results in persistent or significant disability/incapacity, 5) is a congenital anomaly/birth defect, 6) is a medically important event or reaction.

10 To determine the appropriate category, the user should first establish, whether a reported event meets the criteria for the lowest applicable level of diagnostic certainty. If the lowest applicable level of diagnostic certainty of the definition is met, and there is evidence that the criteria of the next higher level of diagnostic certainty are met, the event should be classified in the next category. This approach should be continued until the highest level of diagnostic certainty for a given event could be determined. If the lowest level of the case definition is not met, it should be ruled out that any of the higher levels of diagnostic certainty are met and the event should be classified in additional categories four or five.

11 If the evidence available for an event is insufficient because information is missing, such an event should be categorized as “Reported Respiratory Distress in the Neonate with insufficient evidence to meet the case definition”.

12 An event does not meet the case definition if investigation reveals a negative finding of a necessary criterion (necessary condition) for diagnosis. Such an event should be rejected and classified as “Not a case of Respiratory Distress in the Neonate”.

13 Use of this document should preferably be referenced by referring to the respective link on the Brighton Collaboration website (http://www.brightoncollaboration.org).
