# Supplementary material for: Respiratory distress in the neonate: Case definition & guidelines for data collection, analysis, and presentation of maternal immunization safety data
Source: Vaccine. 2017 Dec 4;35(48Part A):6506–17. doi: 10.1016/j.vaccine.2017.01.046 (PMC5710987; doi:10.1016/j.vaccine.2017.01.046)
Supplement: Supplementary data 1 [file mmc1.docx]

# APPENDIX A: Assessment tools to aid identification of respiratory distress in the neonate.

**Table 4. World Health Organization Classification of Breathing Difficulty**^27^

| **Respiratory rate (breaths/min)** | **Grunting or Chest Indrawing** | **Classification** |
| --- | --- | --- |
| More than 90 | Present | Severe |
| More than 90 | Absent | Moderate |
| 60 to 90 | Present | Moderate |
| 60 to 90 | Absent | Mild |

**Table 5. ACoRN Respiratory Score**^11^

| **Variable** | **Score** | | |
| --- | --- | --- | --- |
|  | **0** | **1** | **2** |
| Respiratory rate | 40 to 60 | 60 to 80 | >80 |
| Oxygen requirement | None | </= 50% | >50% |
| Retractions | None | Mild to moderate | Severe |
| Grunting | None | With stimulation | Continuous at rest |
| Breath sounds on auscultation | Easily heard throughout | Decreased | Barely heard |
| Prematurity | >34 weeks | 30 to 34 weeks | <30 weeks |
| Score is sum of 6 individual elements; Mild: 0 to 4; Moderate: 5 to 8; Severe: >8 or intubated at admission. | | | |

**Table 6. Silverman Scoring System^15,28,29^**

| \| **Variable** \| **Score** \| \| \| \| --- \| --- \| --- \| --- \| \| **0** \| **1** \| **2** \| \| Upper chest retractions \| Synchronized \| Lag on inspiration \| See-saw \| \| Lower chest retractions \| No retractions \| Just visible \| Marked \| \| Xiphoid retractions \| None \| Just visible \| Marked \| \| Nasal dilatation \| None \| Minimal \| Marked \| \| Grunt \| None \| Stethoscope only \| Naked ear \| \| Sum of 5 individual elements; Score >6 is indicative of impending respiratory failure. \| \| \| \|   **Table 7. Downes RDS Score**^15,30^   \| **Variable** \| **Score** \| \| \| \| --- \| --- \| --- \| --- \| \| **0** \| **1** \| **2** \| \| Cyanosis \| None \| In room air \| In 40% FiO2 \| \| Retractions \| None \| Mild \| Severe \| \| Grunting \| None \| Audible with stethoscope \| Audible without stethoscope \| \| Air entry (crying) \| Clear \| Delayed or decreased \| Barely audible \| \| Respiratory rate (min) \| 60 \| 60-80 \| >80 or apneic episodes \| \| Sum of the 5 individual elements; Score >7 is indicative of impending respiratory failure. \| \| \| \| |
| --- | --- | --- | --- | --- | --- | --- | --- | --- | --- | --- | --- | --- | --- | --- | --- | --- | --- | --- | --- | --- | --- | --- | --- | --- | --- | --- | --- | --- | --- | --- | --- | --- | --- | --- | --- | --- | --- | --- | --- | --- | --- | --- | --- | --- | --- | --- | --- | --- | --- | --- | --- | --- | --- | --- | --- | --- | --- | --- | --- | --- | --- | --- |
