# Supplementary material for: Respiratory distress in the neonate: Case definition & guidelines for data collection, analysis, and presentation of maternal immunization safety data
Source: Vaccine. 2017 Dec 4;35(48Part A):6506–17. doi: 10.1016/j.vaccine.2017.01.046 (PMC5710987; doi:10.1016/j.vaccine.2017.01.046)
Supplement: Supplementary data 2 [file mmc2.docx]

# APPENDIX B: Assessment tools to aid identification of general neonatal illness.

**Table 8. Sick Neonate Score (SNS)**^31^

| **Variable** | **Score** | | |
| --- | --- | --- | --- |
|  | **0** | **1** | **2** |
| Respiratory effort | Apnea or Grunting | Tachypnea (>60/min) with or without retractions | Normal (40-60/min) |
| Heart rate | Bradycardia/Asystole | Tachycardia (>160/min) | Normal (100-160/min) |
| Mean blood pressure (mmHg) | <30 | 30-39 | >39 |
| Axillary temperature (˚C) | <36 | 36-36.5 | 36.5-37.5 |
| Capillary filling time (s) | >5 | 3-5 | <3 |
| Random blood sugar (mg/dl) | <40 | 40-60 | >60 |
| SpO_2_ (in room air) | <85% | 85-92% | >92% |
| Sum of the 7 individual elements; Score ≤ 8 is predictive of mortality | | |  |

**Table 9. SNAP II^32^**

| **Variable** | **Value** | **Points** |
| --- | --- | --- |
| Mean Blood Pressure | ≥ 30mmHg | 0 |
|  | 20 - 29mmHg | 9 |
|  | < 20mmHg | 19 |
| Temperature | >35.6˚C | 0 |
|  | 35 - 36.5˚C | 8 |
|  | < 35˚C | 15 |
| PO_2_ (mmHg) / FiO_2_ (%) | > 2.49 | 0 |
|  | 1.0 - 2.49 | 5 |
|  | 0.3 - 0.99 | 16 |
|  | < 0.3 | 28 |
| Lowest serum pH | ≥ 7.2 | 0 |
|  | 7.1 - 7.19 | 7 |
|  | < 7.1 | 16 |
| Multiple Seizures | No | 0 |
|  | Yes | 19 |
| Urine Output (ml/kg/hr) | ≥ 1 | 0 |
|  | 0.1 - 0.9 | 5 |
|  | < 0.1 | 18 |
| Score and severity of illness: mild: 1-20, moderate: 21-40, severe >40 | | |

**Figure 1. Neonatal Trigger Score (NTS)^33^**

| **Variable** | | **Hours from Time of Birth** | | | | | | | |  |  |
| --- | --- | --- | --- | --- | --- | --- | --- | --- | --- | --- | --- |
|  |  | Birth | 1hr | 2hr | 4hr | 6hr | 8hr | 10hr | 12hr |  | **Score** |
| Temperature (˚C) | > 38.0 |  |  |  |  |  |  |  |  |  | 0 |
|  | 37.5 - 38.0 |  |  |  |  |  |  |  |  |  | 1 |
|  | 36.5 - 37.4 |  |  |  |  |  |  |  |  |  | 2 |
|  | 36.0 - 36.4* |  |  |  |  |  |  |  |  |  | 3 |
|  | < 36.0 |  |  |  |  |  |  |  |  |  |  |
| Heart rate (beats/min) | > 220 |  |  |  |  |  |  |  |  |  |  |
|  | 180 - 219 |  |  |  |  |  |  |  |  |  |  |
|  | 160 - 179 |  |  |  |  |  |  |  |  |  |  |
|  | 100 - 159 |  |  |  |  |  |  |  |  |  |  |
|  | 80 - 99 |  |  |  |  |  |  |  |  |  |  |
|  | < 80 |  |  |  |  |  |  |  |  |  |  |
| Respiratory rate (breaths/min) | > 70 |  |  |  |  |  |  |  |  |  |  |
|  | 51 - 70 |  |  |  |  |  |  |  |  |  |  |
|  | 31 - 50 |  |  |  |  |  |  |  |  |  |  |
|  | 20 - 30 |  |  |  |  |  |  |  |  |  |  |
|  | < 20 |  |  |  |  |  |  |  |  |  |  |
| Respiratory distress | Present |  |  |  |  |  |  |  |  |  |  |
|  | Absent |  |  |  |  |  |  |  |  |  |  |
| Conscious level | Alert / sleeping |  |  |  |  |  |  |  |  |  |  |
|  | Irritable / lethargic / jittery |  |  |  |  |  |  |  |  |  |  |
|  | Unresponsive |  |  |  |  |  |  |  |  |  |  |
| Pre-feed blood sugar (mmol) | > 6.0 |  |  |  |  |  |  |  |  |  |  |
|  | 2.0 - 5.9 |  |  |  |  |  |  |  |  |  |  |
|  | 1.1 - 1.9 |  |  |  |  |  |  |  |  |  |  |
|  | < 1.0 |  |  |  |  |  |  |  |  |  |  |
| *Warm baby / skin-to-skin contact - repeat temperature measurement in 1 hr | | | | | | | | | |  |  |
|  |  |  |  |  |  |  |  |  |  |  |  |
| **Total NTS Score** | | **Action** | | | | | | | | | |
| 0 | | Continue | | | | | | | | | |
| 1 | | Medical review: consider partial septic screen and antibiotics | | | | | | | | | |
| 2 | | Urgent medical review: consider admission to NICU | | | | | | | | | |
| Any observation in this color | | Strongly consider cardiac arrest | | | | | | | | | |
